# The Roles of Phytohormones in Plant Defense Mechanisms Against the Brown Planthopper

**DOI:** 10.3390/genes15121579

**Published:** 2024-12-08

**Authors:** Huiying Wang, Wenjun Zha, An Huang, Yan Wu, Shaojie Shi, Lei Zhou, Aiqing You

**Affiliations:** 1 Laboratory of Crop Molecular Breeding, Ministry of Agriculture and Rural Affairs, Hubei Key Laboratory of Food Crop Germplasm and Genetic Improvement, Food Crops Institute, Hubei Academy of Agricultural Sciences, Wuhan 430064, China; 2 Hubei Hongshan Laboratory, Wuhan 430070, China

**Keywords:** BPH, rice, plant hormone, resistance, stress

## Abstract

The brown planthopper (BPH; *Nilaparvata lugens* Stål) is the most significant insect pest compromising rice production globally. Phytohormones, which are small organic compounds produced by plants, play a crucial role in regulating plant growth and development. Nevertheless, extensive research has established that phytohormones are essential in modulating plant defense against BPH. Plants can achieve equilibrium between growth and defense by utilizing the intricate network of phytohormone signaling pathways to initiate optimal and efficient defensive responses to insects. In this review, we primarily address the roles of phytohormones in conferring resistance against BPH, with a focus on hormone cross-talk. We also discuss the potential value of integrating hormones with other agricultural practices to enhance plant defense and agricultural yield, which highlights the significance of novel approaches for environment-friendly insect pest management.

## 1. Introduction

Rice (*Oryza sativa*) is one of the most crucial crops in the world. Due to its sovereignty, nutritional value, and energy value, half of the global population serves rice as a staple food. Most rice grains are cooked into mature rice, while a small portion is also processed into other food or non-food products [1,2]. However, as sessile organisms, rice plants are constantly threatened by a combination of abiotic and biotic stresses. Insect pest infestations are especially severe due to rice’s preference for growing in warm and humid environments [3]. There are over 100 species that infest rice, but only about 20 species can cause serious loss to the plants [4]. Among them, the BPH is the most widespread, frequent, and destructive pest endangering rice production worldwide [5,6]. These pests use their stylets to extract phloem sap from rice plants, resulting in wilting, lodging, a drop in the proportion of seeds that set, and, potentially, a total cessation of production [7]. Additionally, brown planthoppers transmit numerous bacteria and viruses, including rice grassy stunt virus (RGSV) and rice ragged stunt virus (RRSV), which further jeopardize the safety of rice production [8]. In China, the area damaged by brown planthoppers was estimated to be over 25 million hectares per year, causing a 30% loss in rice yield and ranking first among the various insect pests [6].

**Figure 1:**
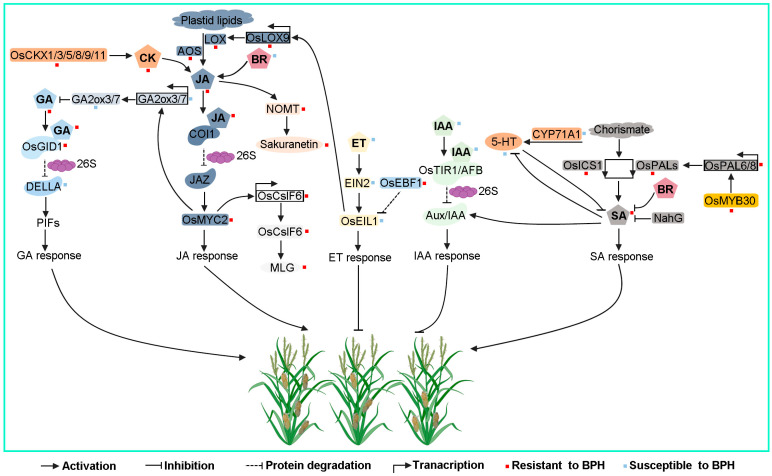
Schematic presentation of potentially conserved hormone cross-talks in resistance to BPH. JA, jasmonic acid; SA, salicylic acid; ABA, abscisic acid; GA, gibberellin; IAA, indole acetic acid; ET, ethylene; BR, brassinosteroid; CK, cytokinin; LOX, lipoxygenase; AOS, allene oxide synthase; COI1, coronatine-insensitive 1; JAZ, JA ZIM-domain; NOMT, naringenin 7-O-methyltransferase; OsCslF6, cellulose synthase-like F6; MLG, mixed-linkage β-1,3;1,4-D-glucans; PAL, phenylalanine ammonium lyase; ICS1, isochorismate synthase 1; 5HT, 5-hydroxytryptamine; NahG, salicylate hydroxylase; GID1, gibberellin-insensitive dwarf 1; GA2ox3, GA 2-oxidase 3; GA2ox7, GA 2-oxidase 7; PIF, phytochrome-interacting factor; TIR1, transport inhibitor response 1; AFB, auxin-signaling F-box; Aux/IAA, auxin/IAA; OsEIL1, ethylene-insensitive 3-like 1; EIN2, ethylene-insensitive 3; OsEBF1, EIN3-binding F-box 1; CKX, cytokinin oxidase/dehydrogenase.

The main strategy for crop protection against insects over the past 50 years has been the application of chemical insecticides, which can quickly kill pests and have the characteristics of easy use and quick effectiveness. However, the long-term and frequent application of pesticides causes brown planthoppers to develop resistance. In addition, pesticide residues have adverse effects on humans and livestock and pollute the environment [4]. Meanwhile, the production of rice cultivars resistant to BPH is perceived as an effective and environmentally sustainable strategy for managing this destructive insect pest. Currently, 17 genes that confer BPH resistance have been cloned and characterized in rice plants [9]. When rice plants are attacked by BPH, numerous signaling molecules involved in plant defense are activated, including calcium ion (Ca^2+^) flux, reactive oxygen species (ROS), mitogen-activated protein kinase, callose, and plant hormones [4,9]. Notably, plant hormones play an essential role in a plant’s response to BPH infestation [10,11]. This review primarily discusses plant hormones’ functions in response to BPH, consequently enhancing the understanding of the relationship between rice and BPH and offering an innovative perspective on more effective environment-friendly pest control methods (Figure 1; Table 1).

## 2. Plant Hormone

Plant hormones are small molecules produced within plants that have a broad range of biological effects, including regulating cell division and elongation, tissue and organ differentiation, flowering, fruiting, maturity, aging, dormancy, germination, and plant immunity [28]. At present, it has been established that various plant hormones, including jasmonate (JA), salicylic acid (SA), gibberellins (GAs), auxin, cytokinin (CK), ethylene (ET), and brassinosteroid (BR), are also involved in the defensive responses to BPH infestation.

### 2.1. Jasmonic Acid

Jasmonic acids are a class of hormones that originate from C18 fatty acids and play vital roles in plant growth and development and defense responses to abiotic and biotic stresses [29,30]. In chloroplasts, membrane lipids are first converted into 12-oxophytodienoic acid (OPDA). Under the actions of the sequential enzymatic reactions of lipoxygenases (LOXs) and allene oxide synthase (AOS), JA is ultimately synthesized [9,10,11]. JA conjugated with isoleucine (JA-Ile) serves as the primary bioactive JA form, catalyzed by JA amido synthetase 1 (JAR1) [31]. In higher plants, JA-Ile is recognized by the receptor coronatine-insensitive 1 (COI1), which mediates the degradation of JAZ (JA ZIM-domain) proteins via the 26S proteasome system. The degradation of JAZ frees the JAZ-suppressed MYC transcription factors, allowing them to activate downstream JA-responsive genes [32,33,34].

JA is a key component of plant defenses against insects, including BPH. Several studies have demonstrated markedly upregulated JA signaling pathways following BPH infestation in both susceptible and resistant backgrounds. And the response is notably stronger in resistant varieties compared with susceptible ones [12,21,35,36,37]. Likewise, JA concentrations are considerably elevated in response to BPH attacks. Exogenous application of methyl jasmonate (MeJA) to rice plants markedly boosted resistance, lowering the survival rate of planthoppers [35]. The assay results indicated that compared with wild-type (WT) plants, the egg-laying rate of BPH was higher in JA-deficient lines (allene oxide cyclase [*OsAOC*]- and *OsMYC2*-knockout lines) [12]. In addition to MeJA and JA-Ile, JA-Val and JA-Leu can also increase resistance to BPH by interacting with COI receptor proteins [38,39]. Furthermore, a multitude of studies have demonstrated that JA signaling can indirectly thwart BPH invasion by controlling the build-up of defense metabolites triggered by BPH. During BPH attacks, the levels of phenolamides, flavonoids, and terpenes were reduced in JA-deficient lines compared with WT plants [12]. Sakuranetin, a flavonoid secondary metabolite, significantly accumulates in regions damaged by BPH. Naringenin 7-O-methyltransferase (*NOMT*) is a key gene in the biosynthesis of flavonoid phytoalexin sakuranetin. In a *NOMT*-knockout mutant created via the CRISPR/Cas-editing system, the ability to synthesize sakuranetin was lost, resulting in an increased susceptibility to the insect. Importantly, a functional JA signaling pathway is crucial for the accumulation of sakuranetin induced by BPH [13]. Mixed-linkage β-1,3;1,4-D-glucans (MLGs) play a significant role in plant–insect interactions [40]. In rice, the overexpression of cellulose synthase-like F6 (*OsCslF6*), a gene involved in MLG synthesis, increases resistance to BPH. Additional investigation revealed that OsMYC2, the main transcription factor in jasmonate signaling, directly binds to the promotor region of *OsCslF6* [14]. A recent study by Chen et al. analyzed multi-omic data from leaves, leaf sheaths, and roots treated with 100 μM of MeJA. They discovered that OsMYC2 regulates early-expressed genes in all tissues and influences the expression of JA repressors and catabolic genes involved in feedback regulation, which suppresses JA signaling and herbivore resistance. Furthermore, in leaf sheaths (i.e., the feeding position of BPH) sprayed with MeJA, a large number of late-expressed genes linked to the biosynthesis of specialized metabolites that protect against BPH were identified [41].

### 2.2. Salicylic Acid

Salicylic acid is a phenolic hormone widely present in plants, which governs plant growth, development, maturity, and aging, as well as plants' resistance to biotic and abiotic stresses. In plants, SA is synthesized through two conserved pathways: the phenylalanine ammonium lyase (PAL) pathway and the isochorismate (IC) pathway. Notably, both pathways utilize chorismate, the end product of the shikimate pathways, as their precursor [42]. Like JA, SA is another conserved positive regulator that plays a significant role in plant resistance to insects. Like JA, BPH infection leads to elevated levels of SA, particularly in resistant varieties [21,35,37,43,44,45]. Additionally, applying exogenous SA also enhances resistance to BPH [35]. Nine *OsPAL* genes were identified in the Nipponbare genome, seven of which were activated in response to BPH attacks, particularly *OsPAL6* and *OsPAL8*. Compared with WT plants, the *OsPAL* RNAi transgenic lines exhibited increased susceptibility, while the *OsPAL8* overexpressing lines showed enhanced resistance under BPH infestation. A similar tendency was observed in the SA accumulation and lignin content [15]. Subsequent investigation revealed that OsMYB30, an R2R3MYB transcription factor, directly binds to the AC-like elements in the promoter regions of *OsPAL6* and *OsPAL8*, thereby regulating their expression and enhancing resistance to the pest [15]. Similar to SA, chorismate serves as a common precursor in the production of serotonin (5-hydroxytryptamine (5HT)) in plants [46]. Lu et al. discovered that SA and serotonin biosynthesis are mutually regulated competitively in rice. Specifically, SA inhibits the expression of the serotonin synthase gene, leading to reduced serotonin production and vice versa. In resistant rice varieties affected by BPH, the expression of the cytochrome P450 gene *CYP71A1*, which encodes tryptamine 5-hydroxylase, is suppressed, resulting in increased SA levels and decreased serotonin levels. Conversely, in susceptible varieties, BPH damage leads to the upregulation of *CYP71A*, causing an increase in serotonin. Research has also shown that elevated serotonin levels in rice promote the growth and development of pests. Consequently, the “arms race” between BPH and rice is partially attributed to the competition between SA and serotonin biosynthesis [16]. Furthermore, plants overexpressing the salicylate hydroxylase gene (*NahG*) were found to be more vulnerable to insect infestations [17].

### 2.3. Gibberellic Acid

GAs influence several developmental processes, including cell expansion and division, which collectively control plant size [47]. When GA is recognized by the gibberellin-insensitive dwarf 1 (GID1) receptor in the nucleus, it interacts with and promotes the degradation of DELLA proteins, the negative regulators of GA signaling, through the action of the E3 ubiquitin ligase–proteasome [47]. In rice, there is only one gene encoding a GA receptor, gibberellin-insensitive dwarf 1 (*OsGID1*), which produces a soluble hormone-sensitive lipase-like protein [48]. Recent research indicates that GA-mediated signaling plays a crucial role in plant defenses against BPH, interacting with JA, SA, and other hormones and defensive secondary metabolites. Following female BPH infestation, the expression of slender rice 1 (*OsSLR1*), which encodes the DELLA protein, was substantially inhibited. In plants overexpressing *OsSLR1*, the expression levels of mitogen-activated protein kinase and WRKY transcripts, as well as the accumulation of JA, ET, and H_2_O_2_, were increased in response to BPH. Conversely, in *OsSLR1*-silenced plants, the levels of defense-related compounds, such as lignin, phenolic acids, and cellulose, were significantly higher, along with increased resistance to BPH [18]. The GA receptor gene *OsGID1* was activated by BPH damage, mechanical injury, and SA treatment but not by JA treatment. In *OsGID1-*overexpressing (*oe-OsGID1*) plants, BPH-induced levels of SA, H_2_O_2_, and three SA pathway-related WRKY transcripts were reduced, while BPH-induced ethylene levels were increased. Bioassays showed that gravid BPH females preferred to infest and lay eggs on WT plants over *oe-OsGID1* plants, with a significantly lower egg-laying rate on *oe-OsGID1* plants. These findings suggest that *OsGID1* positively regulates plant defenses against BPH [19]. Transcriptomic and chemical analyses revealed that BPH-induced damage impedes rice growth by significantly activating the gibberellin metabolic pathway, particularly through upregulating the GA2ox3 and GA2ox7 enzymes. These enzymes convert active GA into inactive forms, contributing to growth inhibition in rice. Additionally, the JA pathway, through its core transcription factor OsMYC2, directly binds to the promoters of *GA2ox3* and *GA2ox7*, thereby modulating the growth inhibition associated with these enzymes [20]. This research has uncovered a novel mechanism for managing the trade-off between plant growth and defense. Specifically, when plants are damaged by BPH, the JA signaling pathway enhances defense responses while simultaneously suppressing plant growth by activating the GA metabolic pathway. This process helps allocate limited resources more efficiently. These findings offer a significant theoretical foundation for developing rice varieties that are both high-yielding and resistant to insects [20].

### 2.4. Auxin

Auxins are a family of endogenous hormones that drive plant growth and development, with indole-3-acetic acid (IAA) being the principal naturally occurring auxin in plants [49]. The main synthetic route for IAA is the indole-3-pyruvate (IPyA) pathway. In this process, tryptophan aminotransferase (TAA) initially converts tryptophan into IPyA, which is subsequently catalyzed into IAA by flavin monooxygenase (FMO) [49]. The *YUCCA* gene encodes the FMO [49]. Auxin is detected by the receptors transport inhibitor response 1 (TIR1) and auxin-signaling F-box (AFB) proteins, leading to the 26S proteasome-mediated degradation of auxin/IAA (Aux/IAA), which are transcriptional repressors of auxin response factors (ARFs). This process enables the activation of auxin response genes [50]. Numerous studies have demonstrated that IAA plays a significant role in plants´ responses to pathogenic bacteria, fungi, and viruses [51,52,53,54,55,56]. In recent studies, IAA was found to play a role in the defense against BPH invasion. Through integrated transcriptomics and metabolomics analyses, Shi et al. discovered that BPH infestation led to significant changes in gene expression related to IAA signaling and synthesis. Most of these differentially expressed genes (DEGs) were downregulated in both Nipponbare and BPH30T (a resistant transgenic line with *Bph30*) plants [57,58], but the extent of this decrease was more pronounced in BPH30T plants than in Nipponbare plants, such as *OsTAA1*, *OsYUC5*, and *OsYUC9* [21]. Measurements revealed that IAA accumulation significantly decreased in BPH30T plants, whereas IAA levels remained unchanged in the susceptible Nipponbare variety. Additionally, IAA treatment was found to weaken the BPH resistance conferred by *Bph30* [21]. Zhang et al. observed that the IAA content in the BPH-resistant variety C331 was lower compared with that in the Nipponbare variety after BPH feeding. Functional analysis illustrated that exogenous IAA treatment reduced BPH resistance in Nipponbare significantly [59]. These findings suggest that IAA increases susceptibility to BPH in both resistant and susceptible plant varieties. Nevertheless, the underlying molecular mechanism remains unclear.

### 2.5. Cytokinin

CKs govern a variety of physiological functions, most notably plant cell division [60]. The homeostasis of CKs is maintained by a balance between the catabolic enzyme cytokinin oxidase/dehydrogenase (CKX) and the synthetic enzyme adenosine phosphate isopentenyl transferase (IPT) [60]. In CK signaling in plants, there are two types of CK response regulators (RRs) that play central roles: type-A RRs typically act as negative regulators, while type-B RRs generally function as positive regulators [61,62]. In rice, BPH infestation leads to increased CK accumulation and activation of the CK pathway [22,35]. A qRT-PCR assay demonstrated that BPH feeding induced the expression of CK synthetic genes, *OsIPTs*, and type-B response regulator-encoding genes, while the expressions of CK inactivation regulators, namely, CKXs, were reduced [22]. Additionally, treatments with exogenous CKs enhanced resistance to BPH [22,35]. The knockout of each gene—*OsCKX1*, *OsCKX3*, *OsCKX5*, *OsCKX8*, *OsCKX9*, and *OsCKX11*—resulted in enhanced BPH resistance [63]. Further analysis indicated that CK treatment elevates the transcript levels of JA biosynthesis and JA signaling, with minimal changes in the expression of SA pathway-related genes, suggesting that CK-mediated resistance to BPH relies on the JA pathway rather than the SA pathway [22].

### 2.6. Ethylene

ET is a gaseous phytohormone crucial for plant growth and defense responses to biotic stress, including resistance to BPH [10,63,64]. Previous research has shown that silencing the gene *OsERF3* (ethylene-responsive factor 3) enhances resistance to BPH, while overexpressing *OsERF3* increases susceptibility [23]. Furthermore, compared with ET-deficient *as-acs* plants (with antisense expression of ACC synthase 2 [*OsACS2*]), insects exhibited a preference for infesting and laying eggs on WT plants [24]. According to Ma et al., ethylene-insensitive 3-like 1 (OsEIL1), a key positive regulator downstream of ethylene-insensitive 2 (EIN2) in the ET pathway, negatively influences BPH resistance. The F-box protein OsEBF1 interacts with *OsEIL1* and facilitates its degradation, thereby playing a positive role in enhancing BPH resistance [25]. Transcriptome analysis revealed a significant reduction in *OsLOX9* (a JA biosynthetic pathway-related gene) in the *oseil1* mutant. Biochemical analyses showed that OsEIL1 directly interacts with the promoter region of *OsLOX9*, acting as a positive regulator of its expression. These findings denote a negative cross-talk between the JA and ET pathways in response to a BPH attack [25]. *OsEBF2*, homologous to *OsEBF1*, also interacts directly with *OsEIL1* and positively regulates BPH resistance [26]. Recent studies have reported that dim light (DL) reduces insect resistance. Further analysis indicated that the reduced BPH resistance under DL was due to elevated ET biosynthesis and signaling, mediated by phytochrome B (*OsPHYB*) [65]. Exogenous treatment with the ET precursor 1-aminocyclopropane-1-carboxylic acid (ACC) was observed to negatively regulate BPH resistance [65].

### 2.7. Brassinosteroids

BRs are phytohormones that regulate myriad plant growth and development processes [66]. It is established that BR signaling acts as a positive/negative regulator of plant defenses through interactions with multiple other hormone pathways [67,68,69]. For example, BRs negatively regulate rice resistance against the oomycete *Pythium graminicola* (*P. graminicola*)by impacting the SA and GA signaling pathways, either directly or indirectly [67,68]. High-throughput sequencing showed that the JA pathway was upregulated, while the BR pathway was downregulated in plants infected by the rice black-streaked dwarf virus (RBSDV). JA-mediated immunity was found to inhibit BR-mediated susceptibility [69]. Despite this, there is limited understanding of the role of these pathways in the rice–BPH interaction. qRT-PCR analysis indicated that the BR signaling pathway was suppressed following BPH infestation, like BR-insensitive 1 (BRI1) and brassinazole-resistant 1 (BZR1) [17]. Additionally, susceptibility increased significantly in *slg-D* (BR-overproducing mutant), *m107* (BR-excessive mutant), and plants pre-treated with 24-epibrassinolide (BL), whereas BR-deficient mutants, *lhdd10*, exhibited greater resistance compared with WT plants [17,27]. Furthermore, BRs were found to quell the expression of genes associated with the SA pathway and lower SA content while increasing the expression of genes related to the JA pathway and JA content. Notably, the BR-induced suppression of the SA pathway was disrupted in the JA mutants *og1* and *coi1-18*. These results established that BRs enhance rice plants' susceptibility to BPH by modulating the SA and JA pathways [17].

## 3. Perspectives

A multitude of studies have collectively demonstrated the substantial role of phytohormones in resisting BPH invasion. Among these, JA and SA are the primary hormones involved in insect resistance, while other hormones typically exert their effects through interactions with these two (Figure 1; [70,71]). This understanding is supported by the swift advancements in analyzing plant genomes, transcriptomes, and proteomes, which have allowed us to promptly identify key signaling pathways activated during BPH attacks [72]. In a similar vein, plant hormones are essential for managing plant growth and crop yield [73,74,75]. These findings suggest that phytohormones could serve as promising biopesticides in the market due to their potential for financial benefits, nutritional advantages, and environmental sustainability. As such, they could be important contributors to the advancement of sustainable agriculture [70]. Phytohormone signaling pathways allow plants to initiate effective defense mechanisms against insects and regulate growth. By manipulating hormone levels through RNA interference (RNAi), CRISPR/Cas, overexpression, and/or their combinations, researchers can engineer plants to adjust the expression of enzymes or regulatory genes involved in phytohormone metabolism. This approach may help balance defense and growth, presenting a promising area for future research [70]. In conclusion, assimilating phytohormone treatments with agricultural techniques, like cultural management, trap cropping, crop rotation, and biological control, could help develop a sustainable, tridimensional defense system against BPH populations. However, how these factors work together to achieve synergistic results still requires further exploration in the future.

## Figures and Tables

**Table 1:** Roles of phytohormone-related genes in defenses against BPH.

Gene Name	Annotation	Function in BPH Resistance	Reference
*OsAOC*	Allene oxide cyclase for JA synthesis	The content of JA was decreased in the *OsAOC*-knockout lines, but the hatching rate of BPH in the *OsAOC*-knockout lines was increased.	[12]
*OsMYC2*	MYC transcription factor in JA signaling pathway	The hatching rate of BPH in the *OsMYC2*-knockout lines was increased.	[12]
*NOMT*	Naringenin 7-O-methyltransferase for JA-related sakuranetin synthesis	Sakuranetin accumulated greatly in the area damaged by BPH. The BPH-elicited accumulation of sakuranetin depended on intact JA signaling transduction. The *NOMT*-knockout mutant showed more susceptibility to the insect.	[13]
*OsCslF6*	MLG synthetase	Overexpressing *OsCslF6* enhanced BPH resistance. OsMYC2 bound to the promotor region of *OsCslF6* directly.	[14]
*OsPAL6*	Phenylalanine ammonium lyase for SA synthesis	Compared with the WT, the RNAi transgenic lines showed more susceptibility, while the overexpressing lines exhibited more resistance.	[15]
*OsPAL8*	Phenylalanine ammonium lyase for SA synthesis	Compared with the WT, the RNAi transgenic lines showed more susceptibility, while the overexpressing lines exhibited more resistance.	[15]
*OsMYB30*	MYB transcription factor in SA signaling pathway	Interacted with the promotor of *OsPAL6* and *OsPAL8*.	[15]
*CYP71A1*	Cytochrome P450 for serotonin synthesis	SA and serotonin are from the common precursor chorismate, so there is a mutual competitive regulation between the biosynthesis of SA and serotonin in rice. Upon damage by BPH, the expression of *CYP71A1* was suppressed, resulting in a decrease in serotonin accumulation but an increase in SA accumulation in the resistant plants, while in the susceptible plants, the opposite result was observed.	[16]
*NahG*	SA hydroxylase gene	Compared with the WT, the transgenic lines overexpressing *NahG* exhibited more susceptibility to BPH infestation.	[17]
*OsSLR1*	GA DELLA protein	In the *OsSLR1*-overexpressing lines, the BPH-elicited expression levels of the mitogen-activated protein kinase and WRKY transcripts, as well as the accumulation of JA, ET, and H_2_O_2_, were increased. Meanwhile, in *OsSLR1-*silenced plants, the contents of defense-related compounds, such as lignin, phenolic acids, and cellulose, were greatly enhanced, as well as the resistance to BPH.	[18]
*OsGID1*	GA receptor gibberellin-insensitive dwarf 1	In the plants overexpressing OsGID1 (oe-GID1), the BPH-elicited levels of SA, H_2_O_2_, and three SA pathway-related WRKY transcripts were reduced; however, the BPH-induced level of ethylene was enhanced. Bioassays revealed that the gravid BPH females preferred to infest and lay eggs on the WT plants than on the oe-GID1 plants.	[19]
*GA2ox3*	GA 2-oxidase for GA metabolism	The expression of *GA2ox3* was activated by BPH infestation. Further analysis showed that OsMYC2 bound to the promoter region of *GA2ox3.*	[20]
*GA2ox7*	GA 2-oxidase for GA metabolism	The expression of *GA2ox7* was activated by BPH infestation. Further analysis showed that OsMYC2 bound to the promoter region of *GA2ox7*.	[20]
*OsTAA1*	Tryptophan aminotransferase	The expression of *OsTAA1* was inhibited by BPH infestation. The degree of decrease was greater in resistant BPH30T plants than that in susceptible Nipponbare plants.	[21]
*OsYUC5*	Flavonoid monooxygenase for IAA synthesis	The expression of *OsYUC5* was inhibited by BPH infestation. The degree of decrease was greater in resistant BPH30T plants than that in susceptible Nipponbare plants.	[21]
*OsTAA9*	Flavonoid monooxygenase for IAA synthesis	The expression of *OsYUC9* was inhibited by BPH infestation. The degree of decrease was greater in resistant BPH30T plants than that in susceptible Nipponbare plants.	[21]
*OsCKX1*	CK oxidase/dehydrogenase	Knockout lines showed enhanced BPH resistance.	[22]
*OsCKX3*	CK oxidase/dehydrogenase	Knockout lines showed enhanced BPH resistance.	[22]
*OsCKX5*	CK oxidase/dehydrogenase	Knockout lines showed enhanced BPH resistance.	[22]
*OsCKX8*	CK oxidase/dehydrogenase	Knockout lines showed enhanced BPH resistance.	[22]
*OsCKX9*	CK oxidase/dehydrogenase	Knockout lines showed enhanced BPH resistance.	[22]
*OsCKX11*	CK oxidase/dehydrogenase	Knockout lines showed enhanced BPH resistance.	[22]
*OsERF3*	ET-responsive factor 3	Silencing *OsERF3* enhanced resistance against BPH, and overexpressing *OsERF3* produced more susceptibility to BPH.	[23]
*OsACS2*	ACC synthase for ET synthesis	*as-acs* plants showed enhanced BPH resistance.	[24]
*OsEIL1*	Key positive regulator in ET pathway	OsEIL1 negatively regulated BPH resistance. OsEIL1 was the positive regulator of *OsLOX9* expression, which indicated the negative cross-talk of the JA and ET pathways in response to BPH attack.	[25]
*OsEBF1*	EIN3-binding F-box protein in ET pathway	OsEBF1 played a positive role in BPH resistance.	[25]
*OsEBF2*	EIN3-binding F-box protein in ET pathway	OsEBF2 played a positive role in BPH resistance.	[26]
*BRI1*	BR receptor BR-insensitive 1	Its expression was decreased post-BPH infestation.	[17]
*BZR1*	BR signaling component brassinazole-resistant 1	Its expression was decreased post-BPH infestation.	[17]
*SLG*	BAHD acyltransferase-like protein	BR-overproducing mutant *slg-D*, which overexpressed *SLG*, showed more susceptibility to BPH than WT plants.	[17,27]

## Data Availability

The original contributions presented in this study are included in this article. Data will be made available upon request.

## Abbreviations

BPH: brown planthopper; RGSV: rice grassy stunt virus; RRSV: rice ragged stunt virus; JA: jasmonate; SA: salicylic acid; GA: gibberellin; IAA: indole-3-acetic acid; CK: cytokinin; ET: ethylene; BR: brassinosteroid; OPDA: 12-oxophytodienoic acid; LOXs: lipoxygenases; AOS: allene oxide synthase; JA-Ile: JA conjugated with isoleucine; COI1: coronatine-insensitive 1; JAZ: JA ZIM-domain; MeJA: methyl jasmonate; NOMT: naringenin 7-O-methyltransferase; MLGs: mixed-linkage β-1,3;1,4-D-glucans; OsCslF6: cellulose synthase-like F6; PAL: phenylalanine ammonium lyase; IC: isochorismate; ICS1: isochorismate synthase 1; 5HT: 5-hydroxytryptamine; NahG: salicylate hydroxylase; GID1: gibberellin-insensitive dwarf 1; GA2ox3: GA 2-oxidase 3; GA2ox7: GA 2-oxidase 7; OsSLR1: lender rice 1; PIF: phytochrome-interacting factor; TAA: tryptophan aminotransferase; IPyA: indole-3-pyruvate; FMO: flavin monooxygenase; TIR1: transport inhibitor response 1; AFB: auxin-signaling F-box; Aux/IAA: auxin/IAA; ARFs: auxin response factors; DEGs: differentially expressed genes; BPH30T: a resistant transgenic line with *Bph30*; OsTAA1: tryptophan aminotransferase 1; BRI1: BR-insensitive 1; BZR1: brassinazole-resistant 1; CKX: cytokinin oxidase/dehydrogenase; IPT: isopentenyl transferase; RRs: response regulators; OsERF3: ethylene-responsive factor 3; OsEIL1: ethylene-insensitive 3-like 1; EIN2: ethylene-insensitive 3; DL: dim light; OsPHYB: phytochrome B; ACC: 1-aminocyclopropane-1-carboxylic acid; RBSDV: rice black-streaked dwarf virus.
